# Missed Opportunities for Congenital Syphilis Prevention — Clark County, Nevada, 2017–2022

**DOI:** 10.15585/mmwr.mm7420a3

**Published:** 2025-06-05

**Authors:** Jessica A. Penney, Angel Stachnik, Cheryl Radeloff, Tabby Eddleman, Heidi Laird, Ying Zhang, Cassius Lockett

**Affiliations:** ^1^Epidemic Intelligence Service, CDC; ^2^Division of Disease Surveillance and Control, Southern Nevada Health District, Las Vegas, Nevada.

**Summary**

**What is already known about this topic?**

U.S. syphilis cases approximately tripled during 2018–2022. Congenital syphilis can result in severe infant morbidity and death but is preventable through appropriate screening and treatment of pregnant females.

**What is added by this report?**

During 2017–2022, in Clark County, Nevada, prenatal care was accessed by approximately one half of females who had an infant with congenital syphilis. Approximately one half of these mothers had an emergency department encounter during pregnancy that was a possible opportunity for timely testing; syphilis testing was performed at 68% of these encounters.

**What are the implications for public health practice?**

Lack of prenatal care was a barrier to timely syphilis testing and treatment. Encounters in nontraditional settings such as emergency departments could provide an opportunity for timely testing and, if linked to timely treatment, might help prevent congenital syphilis.

## Abstract

In 2022, Nevada ranked eighth in the United States in incidence of congenital syphilis, a disease that can lead to stillbirth, miscarriage, or neonatal death. Appropriate and timely screening of pregnant females for syphilis and treatment, when indicated, are crucial for preventing congenital syphilis. Southern Nevada Health District (Clark County) disease surveillance data for 2017–2022 were reviewed to identify females of reproductive age (aged 15–44 years) with confirmed or probable syphilis who had a liveborn or stillborn infant with congenital syphilis and to assess their receipt of prenatal care, syphilis testing and, when indicated, syphilis treatment. Clark County emergency department (ED) visit data were reviewed for these females to explore whether ED visits might represent an opportunity to screen pregnant females for syphilis. Among 195 females identified, 43.1% (84) reported receiving prenatal care during pregnancy. Over one half (57.4%) of the females had at least one ED encounter ≥30 days before delivery and had not yet received testing for syphilis at the time of the encounter; syphilis testing was performed at 68.4% of these encounters. Lack of prenatal care was a considerable barrier to timely testing and treatment in Clark County, Nevada. Encounters in nontraditional care settings, including but not limited to EDs, could provide an opportunity for syphilis screening of pregnant females who do not access prenatal care. If linked to timely treatment, such encounters might help prevent congenital syphilis.

## Introduction

Cases of primary and secondary syphilis among females of reproductive age (aged 15–44 years) approximately tripled in the United States during 2018–2022.* Congenital syphilis can lead to stillbirth, miscarriage, or neonatal death, as well as blindness, deafness, developmental delay, and skeletal abnormalities among surviving infants (*1*). Screening for and treatment of syphilis at appropriate times during pregnancy has been indicated to prevent syphilis morbidity in pregnant females and prevent congenital syphilis (*1*,*2*). Missed opportunities for congenital syphilis prevention in the United States have been previously identified (*3*,*4*), with lack of timely testing and inadequate treatment during pregnancy contributing to 88% of congenital syphilis cases reported nationally in 2022 (*3*). In 2022, Nevada ranked eighth in the United States in rates of reported cases of primary and secondary syphilis and congenital syphilis. Clark County is the most populous county in Nevada. Surveillance data on syphilis cases identified in pregnant females during 2017–2022 were analyzed to identify missed opportunities for congenital syphilis prevention in Clark County.

## Methods

### Data Sources and Study Population

Two data sources were used: Southern Nevada Health District (SNHD) disease surveillance data and emergency department (ED) discharge diagnosis data from all hospitals in Clark County. Quarterly ED discharge data from hospitals and intermediate care facilities were obtained from the Center for Health Information Analysis.^†^ Cases meeting the Council of State and Territorial Epidemiologists’ syphilis case definition^§^ among females aged 15–44 years, and reported by electronic laboratory testing results to SNHD during 2017–2022, were included. Pregnancy status was obtained from surveillance data collected during standard disease investigation interviews. To identify associated congenital syphilis cases, a linkage was performed through a unique parent identification variable between mother and 1) stillborn infants with congenital syphilis and 2) liveborn infants who met surveillance criteria for confirmed and probable congenital syphilis. After linkage, the resultant matched dataset was de-identified before analysis.

### Classification of Missed Opportunities for Congenital Syphilis Prevention

To identify potential missed opportunities for congenital syphilis prevention among mothers who delivered an infant with congenital syphilis, a cascading framework of missed prevention opportunities was applied. The cascading framework classified missed opportunities as follows: 1) no reported prenatal care during pregnancy, 2) prenatal care accessed <45 days before delivery, 3) prenatal care accessed ≥45 days before delivery with syphilis testing performed <45 days before delivery, and 4) prenatal care accessed and syphilis testing performed ≥45 days before delivery with treatment initiated <30 days before delivery. A median interval from diagnosis to treatment of 14 days that has been observed for other sexually transmitted infections (*5*) was factored into the definition for timely care.^¶^ Timely care was defined as 45 days before delivery given the time required for the patient to seek care, have appropriate testing performed, receive laboratory results, and start appropriate treatment if recommended. If prenatal care was reported but date of first prenatal visit was not available, the date of first syphilis testing was considered the date of first prenatal care access. Syphilis testing performed outside of the prenatal care setting was also quantified.

### Syphilis Testing During ED Encounters

Because ED visits that occurred during the woman’s pregnancy could be an opportunity for mothers not accessing traditional prenatal care to be tested and treated, a fuzzy match** was performed between SNHD surveillance data and Clark County ED diagnosis data for all cases in which a woman delivered an infant with congenital syphilis. A possible opportunity for testing was defined as an encounter for care in the ED of a woman meeting the following criteria: pregnant at time of encounter, encounter ≥30 days before delivery, had possible syphilis at time of encounter based on surveillance staging,^††^ and did not have previous syphilis testing documented in surveillance data on the date of the ED visit. To determine if pregnancy status was known at the time of ED encounter, ED diagnosis data were reviewed to identify *International Classification of Diseases, Tenth Revision* (ICD-10) codes associated with pregnancy (ICD-10 O00–O9A). This activity was reviewed by CDC, deemed not research, and was conducted consistent with applicable federal law and CDC policy.^§§^

### Data Analysis

Rates of syphilis (all stages^¶¶^) among females aged 15–44 years during 2017–2022 were calculated and expressed as cases per 100,000 population. Descriptive characteristics were stratified by the presence or absence of linkage to a congenital syphilis case. Missed opportunities for prevention were reported as counts and percentages. Analyses were completed using RStudio (version 6.1; RStudio).

## Results

### Syphilis Cases in Reproductive Aged and Pregnant Females

**FIGURE 1:**
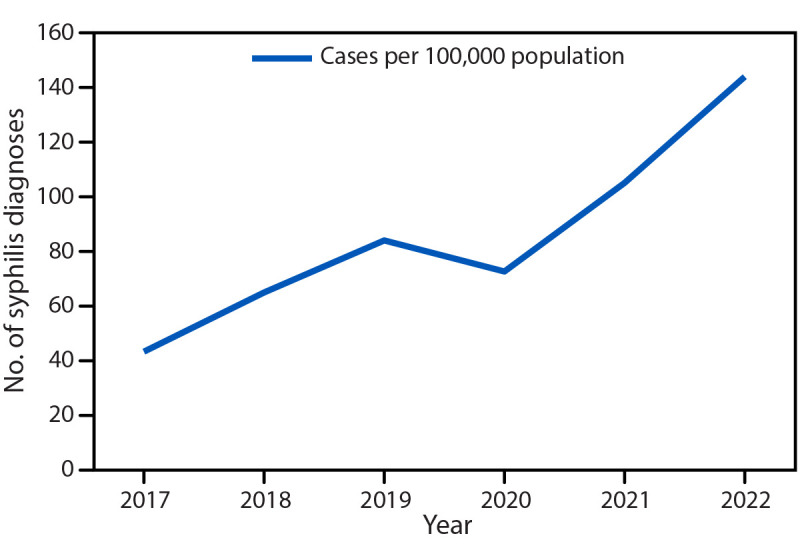
Syphilis diagnoses*^,†^ among females of reproductive age^§^ — Clark County, Nevada, 2017–2022 * Syphilis diagnoses are for all stages. ^†^ Syphilis diagnoses of any stage (e.g., primary, secondary, early nonprimary nonsecondary, and syphilis of unknown duration or late) as defined by the Nationally Notifiable Diseases Surveillance System syphilis case definition were included. Syphilis (*Treponema pallidum*) 2018 Case Definition | CDC ^§^ Females aged 15–44 years.

During 2017–2022 in Clark County, Nevada, the incidence of syphilis (all stages) among reproductive aged females increased from 43.1 to 143.9 per 100,000 population representing a relative increase of 333.9%*** (Figure 1). During this period, 530 pregnant females received a syphilis diagnosis, 195 (36.8%) of whom delivered an infant with congenital syphilis (Table).

**TABLE:** Demographic and clinical characteristics of pregnant females who received a syphilis diagnosis,* by pregnancy outcome — Clark County, Nevada, 2017–2022

Characteristic	Delivered an infant with congenital syphilis, no. (%) n = 195	Did not deliver an infant with congenital syphilis, no. (%) n = 335
Age, yrs, mean (SD)	27.9 (5.7)	26.8 (5.7)
**Race and ethnicity^†^**		
American Indian or Alaskan native	1 (0.5)	2 (0.6)
Asian or Pacific Islander	0 (—)	12 (3.6)
Black or African American	81 (41.5)	154 (46.0)
Hispanic or Latino	39 (20.0)	83 (24.8)
White	66 (33.8)	72 (21.5)
Other/Multiracial	4 (2.1)	6 (1.8)
Unknown	4 (2.1)	6 (1.8)
**Syphilis surveillance staging**		
Primary or secondary syphilis	24 (12.3)	49 (14.6)
Early nonprimary nonsecondary	43 (22.1)	61 (18.2)
Unknown duration or late	130 (66.7)	225 (67.2)
**Prenatal care accessed**		
Yes	84 (43.1)	286 (85.4)
No	111 (56.9)	40 (11.9)
Unknown	0 (—)	9 (2.9)
**Trimester prenatal care first accessed^§^**		
First (wks 1–12)	21 (25.0)	—
Second (wks 13–28)	24 (28.6)	—
Third (wks 29–40)	28 (33.3)	—
Unknown	11 (13.1)	—

* Syphilis diagnoses of any stage (e.g., primary, secondary, early nonprimary nonsecondary, and syphilis of unknown duration or late) as defined by the Nationally Notifiable Diseases Surveillance System syphilis case definition were included. Syphilis (*Treponema pallidum*) 2018 Case Definition | CDC

^†^ Persons of Hispanic or Latino (Hispanic) origin might be of any race but are categorized as Hispanic; all racial groups are non-Hispanic.

^§^ Trimester of first prenatal care access among those who accessed prenatal care was available only for pregnant females associated with a congenital syphilis case. Trimester of care data are collected as part of the congenital syphilis case investigation and are not captured in surveillance data for all pregnant females with a syphilis diagnosis.

### Missed Opportunities for Congenital Syphilis Prevention

**FIGURE 2:**
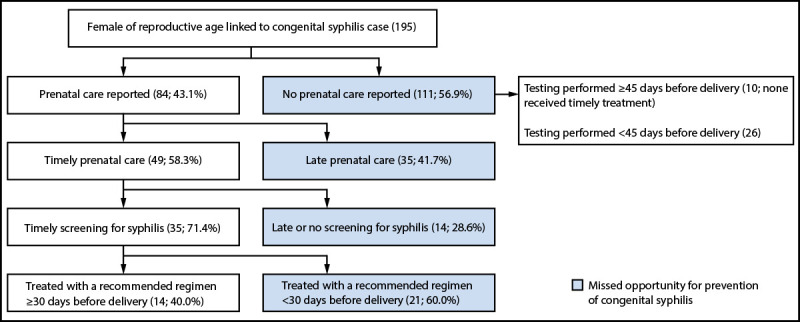
Cascading framework of missed opportunities for congenital syphilis prevention — Clark County, Nevada, 2017–2022*^,†,§^ * Timely is defined as ≥45 days before delivery for prenatal care and screening and ≥30 days before delivery for initiation of appropriate treatment. ^†^ Testing among females with no prenatal care reported included 26 females who received testing <45 days before delivery, but before health care encounter for delivery. ^§^ Females of reproductive age are those aged 15–44 years.

Among 335 mothers with diagnosed syphilis whose pregnancy did not result in an identified case of congenital syphilis, most (85.4%) received prenatal care (Table). Among the 195 mothers who delivered an infant with congenital syphilis, 84 (43.1%) reported having received prenatal care during their pregnancy, with 21 (25.0%) initiating care in the first trimester, 24 (28.6%) in the second trimester, and 28 (33.3%) in the third trimester; information on trimester of prenatal care initiation was missing for 11 (13.1%) mothers. Among the 84 mothers who delivered an infant with congenital syphilis and who reported accessing prenatal care, 49 (58.3%) reported accessing this care ≥45 days before delivery; 35 (71.4%) of these mothers received testing for syphilis ≥45 days before delivery, while the remaining 14 (28.6%) received late or no testing (Figure 2). Among the 35 (17.9%) mothers who delivered an infant with congenital syphilis and who accessed prenatal care and received testing for syphilis ≥45 days before delivery, 14 (40.0%) were treated with a recommended regimen for syphilis ≥30 days before delivery; the remainder were treated with a recommended regimen <30 days before delivery. Among those treated with a recommended regimen ≥30 days before delivery, seven (50.0%) had serologic data available to assess treatment response. All seven had serologic test results compatible with reinfection or treatment failure; five (71.4%) of these mothers were treated for syphilis in their first trimester of pregnancy and did not receive retesting until the time of delivery. Among the 111 mothers who delivered an infant with congenital syphilis and who did not report accessing prenatal care, 36 (32.4%) had syphilis testing documented before the health care encounter for delivery; for most (72.2%) of these mothers, testing was received <45 days before delivery. Of the 10 females who received testing >45 days before delivery, none received timely treatment.

### Syphilis Testing During ED Encounters

Among the 195 mothers who delivered an infant with congenital syphilis, 112 (57.4%) had one or more documented ED visit that met the defined criteria (pregnant at time of the encounter which occurred ≥30 days before delivery, had possible syphilis at time of the encounter based on surveillance staging, and did not receive previous syphilis testing documented in surveillance data on the date of the ED visit) as a possible opportunity for ED syphilis testing, 53 (47%) of whom reported not receiving prenatal care. Of the 112 females, 59 (53%) received testing during an ED visit. A total of 250 ED visits meeting the defined criteria were identified among these 112 females. Positive pregnancy status was documented in the ED medical record in 54 (21.6%) of these visits. Syphilis testing was performed at 171 (68.4%) of these visits.

## Discussion

Lack of timely prenatal care was a considerable barrier to timely testing and treatment for syphilis during pregnancy and subsequent prevention of congenital syphilis in Clark County, Nevada, during 2017–2022. Since 2021, Nevada law has required that health care providers screen pregnant females for syphilis during their first prenatal visit, early in the third trimester of pregnancy (28–32 weeks gestational age), and at the time of delivery.^†††^ However, not all pregnant females access traditional prenatal care. Providing testing in alternative settings for females who do not access prenatal care has been shown to improve timely identification and treatment of persons with syphilis during pregnancy (*4*,^6^–*8*) and might prevent congenital syphilis. For example, novel interventions have been piloted to increase syphilis screening among at-risk populations during ED encounters (e.g., providing routine screening of patients meeting defined criteria, with the option to “opt-out” based on patient declination or provider documentation that the patient is not a candidate for screening during the encounter) (*6**,**9*). In this analysis, approximately one half of mothers who delivered an infant with congenital syphilis had at least one ED visit that might have represented an opportunity for syphilis testing; however, testing was not performed at approximately one third of these encounters. Further, approximately one third of mothers who delivered an infant with congenital syphilis and who did not access prenatal care received testing for syphilis during pregnancy, although testing occurred <45 days before delivery in approximately 70% of these cases, and none received timely treatment. These data suggest an opportunity might exist to improve prevention of congenital syphilis in Clark County by offering syphilis testing to reproductive-age persons during health care visits, including at EDs, and by ensuring linkage to appropriate treatment for those who receive a positive test result.

### Limitations

The findings in this report are subject to at least four limitations. First, case data included in this analysis include only syphilis diagnoses reported in one county in Nevada, and findings might not be generalizable to all populations. However, surveillance data used for this analysis represent complete laboratory testing performed for Clark County residents, and Clark County constitutes the majority of syphilis morbidity in the state, representing approximately 78% of the primary and secondary syphilis cases reported in 2021. Second, surveillance data contain limited information regarding a number of individual- and community-level factors that could influence congenital syphilis prevention, such as access to prenatal care, lack of transportation, and health insurance status. Third, data were not available on the results of syphilis testing received in the ED or on treatment of those females who received positive test results. To prevent congenital syphilis, it is imperative that systems are in place to ensure that test results are reviewed and that females who received positive test results are linked to timely and appropriate treatment. Finally, the analysis did not include reasons for ED encounters, and the treating clinician might have determined that a patient was not a candidate for syphilis testing at a given encounter.

### Implications for Public Health Practice

Lack of access to timely prenatal care was a major barrier to congenital syphilis prevention in Clark County, and efforts should be made to improve access to prenatal care. In addition, in high-prevalence settings such as Clark County, opt-out syphilis testing of females of reproductive age during any health-related encounter, such as at appropriate ED visits or in other nontraditional care-related settings, might increase timely testing of pregnant females who are unable or unlikely to access prenatal care. If systems are in place to ensure follow-up for timely and appropriate treatment of females who received positive test results, this approach could help prevent cases of congenital syphilis.

## Footnotes

* STI Statistics | CDC

† Nevada Healthcare Quarterly Reports (NHQR) (CHIA) - NRHP Reporting and Data

§
Syphilis (*Treponema pallidum*) 2018 Case Definition | CDC

¶ Timely care was defined as 45 days before delivery given the time required for patient to seek care, have appropriate testing performed, receive laboratory results, and start appropriate treatment if recommended.

** Fuzzy matching is a technique to link persons across data sources. Typically, this uses numerous fields which combine to make an approximate match, and for this analysis, a unique variable was created using letters from first name, last name, and digits from date of birth.

†† Estimated incubation period based on surveillance staging assigned during investigation. Syphilis |Pocket Guide for Providers | CDC

§§ 45 C.F.R. part 46.102(l)(2), 21 C.F.R. part 56; 42 U.S.C. Sect. 241(d); 5 U.S.C. Sect. 552a; 44 U.S.C. Sect. 3501 et seq.

¶¶ Syphilis diagnoses of any stage (e.g., primary, secondary, early nonprimary nonsecondary, and syphilis of unknown duration or late) as defined by the Nationally Notifiable Diseases Surveillance System syphilis case definition were included.

*** Population estimate based on 2022 American Community Survey population data for Clark County, Nevada.

†††
NRS: Chapter 442 – Maternal and Child Health; Abortion
